# Smaller Optic Discs Show Higher Macular Flow Density: An Optical Coherence Tomography Angiography Study

**DOI:** 10.3390/biomedicines14061387

**Published:** 2026-06-19

**Authors:** Charlotte Egbring, Sarah Kleemann, Moritz Fabian Danzer, Nicole Eter, Jens Julian Storp

**Affiliations:** 1 Department of Ophthalmology, University of Muenster Medical Center, 48149 Muenster, Germany; sarah.kleemann@med.uni-muenchen.de (S.K.); nicole.eter@ukmuenster.de (N.E.); jensjulian.storp@ukmuenster.de (J.J.S.); 2 Institute of Biostatistics and Clinical Research, University of Muenster, 48149 Muenster, Germany; moritzfabian.danzer@ukmuenster.de

**Keywords:** optical coherence tomography angiography, macular flow density, superficial capillary plexus, deep capillary plexus, foveal avascular zone, Bruch’s membrane opening area, optic disc size

## Abstract

**Background/Objectives**: This study aimed to investigate the correlation between macular flow density (FD) as well as foveal avascular zone (FAZ) characteristics and optic disc size, quantified by Bruch’s membrane opening area (BMOA). In addition, potential differences in FD and FAZ parameters among optic disc size cohorts were evaluated. **Methods**: In this retrospective, single-centre study, 151 eyes from 151 healthy participants examined at the University Hospital Münster, Germany, were included. Each subject underwent macular and optic disc optical coherence tomography angiography (OCT-A). Rank correlation coefficients for clustered data were computed to assess associations between FD values and BMOA. Further analyses compared FD and FAZ parameters among three optic disc size groups based on their quantiles. **Results**: Statistical analysis revealed a significant negative correlation between FD in several macular subsectors and BMOA. When stratified by optic disc size, FD in the superficial capillary plexus (SCP) was significantly higher in eyes with the smallest discs compared with intermediate ones, and FD in the deep capillary plexus (DCP) was significantly higher in intermediate discs compared with the largest group. Additionally, both SCP and DCP showed higher absolute FD values in eyes with the smallest optic discs compared with those with the largest. No significant group differences were detected for foveal FD, FAZ area, or FAZ perimeter. **Conclusions**: This study contributes to normative OCT-A data by incorporating optic disc size as a variable. While FAZ parameters appeared independent of BMOA, eyes with smaller optic discs demonstrated higher FD values in both SCP and DCP.

## 1. Introduction

Optical coherence tomography (OCT) is a non-invasive diagnostic imaging technology introduced in 1991 that enables in vivo cross-sectional visualization of the retina [1]. It provides high-resolution, quantitative assessment of individual retinal layers, such as the peripapillary retinal nerve fibre layer (RNFL) and the macular ganglion cell layer thickness (mGCLT), both of which serve as structural proxies for the number and integrity of retinal ganglion cells [2,3,4,5]. Over the past decades, OCT has become an indispensable tool in ophthalmology for the diagnosis, monitoring, and differentiation of various optic neuropathies and retinal diseases [6,7,8].

Previous investigations have yielded conflicting results regarding the relationship between the thickness of individual retinal layers and optic disc area. While some studies have reported a positive correlation between RNFL thickness and optic disc area [9,10], others contrarily describe no significant association between these parameters [11,12]. Histological studies have demonstrated a quantitative dependence of retinal ganglion cell count on optic disc area [13]. Conversely, other authors have shown that mGCLT appears to be independent of optic disc area [14].

More recently, OCT angiography (OCT-A) has emerged as an advanced, non-invasive imaging modality that allows simultaneous visualization of retinal microvasculature and cross-sectional retinal architecture [15]. OCT-A provides high-resolution en face representations of different vascular plexuses, such as the superficial capillary plexus (SCP)—a network of vessels within the inner retinal layers encompassing the RNFL and GCL—and the deep capillary plexus (DCP), which supplies oxygen and nutrients primarily to bipolar cells and photoreceptors [16]). Although OCT-A has been extensively applied to characterize pathological changes in conditions such as diabetic retinopathy, retinal venous and arterial occlusions, uveitis, and age-related macular degeneration [17], the influence of physiological and anatomical variables on OCT-A metrics remains less well understood. Among these factors, optic disc size may represent a key determinant of retinal perfusion patterns.

In clinical practice, optic discs are commonly categorized as small, medium, or large, a distinction that can assist in the identification of risk factors associated with optic nerve head (ONH) morphology. For instance, small discs have been linked to an increased risk of anterior ischemic optic neuropathy (AION) [18], whereas distinguishing large optic discs from glaucomatous optic neuropathy remains challenging due to the appearance of pronounced cupping [19].

The documentation and classification of optic disc size have traditionally relied on fundus photography, developed in the early 20th century. The introduction of the confocal microscope and the subsequent development of confocal scanning laser ophthalmoscopy (CSLO) laid the foundation for the Heidelberg Retinal Tomograph (HRT), which generates three-dimensional topographic maps for calculating optic disc parameters. Since then, the HRT has been widely regarded as a standard instrument for ONH analysis, particularly in glaucoma diagnostics [20], featuring a well-established classification system for micro-, normo- and macrodiscs. In recent years, however, studies using OCT have demonstrated that measurements based on Bruch’s membrane opening area (BMOA) offer a reliable and reproducible approach for characterizing optic disc morphology, resulting in the application of OCT technology rather than CSLO in the majority of current research and daily clinical practice [21,22,23,24]. However, to date, a universally accepted categorization or threshold definition for micro- and macrodiscs in OCT-based imaging remains lacking.

The primary aim of the present study is to analyze macular flow density (FD) across SCP and DCP, as well as foveal avascular zone (FAZ) characteristics, in relation to optic disc size defined by BMOA in a healthy population. Additionally, FD and FAZ parameters will be compared across optic disc size groups to evaluate the diagnostic relevance of disc morphology. Finally, the data obtained in this study may serve as a normative reference for macular FD and FAZ parameters across the spectrum of optic disc sizes, thereby improving the interpretability of OCT-A findings in both research and clinical practice.

## 2. Materials and Methods

This retrospective study was approved by the Ethics Committee of the Medical Association of Westphalia-Lippe in collaboration with the University of Münster (reference number: 2024-332-f-S). All procedures adhered to the ethical standards outlined in the Declaration of Helsinki. Participants were healthy Caucasian volunteers who attended the Department of Ophthalmology at the University of Münster Medical Centre between 01/2021 and 09/2025. The requirement for written informed consent was waived, as the data analyzed in this study were collected as part of routine clinical care. All data were anonymized prior to analysis to ensure patient confidentiality.

All subjects underwent a comprehensive and standardized ophthalmological assessment, including anterior segment inspection, fundus evaluation, refraction testing, and measurement of intraocular pressure. Exclusion criteria comprised high myopia (defined as a spherical equivalent of −6 diopters or less), any signs of retinal pathology, or optic nerve disease. Additional exclusion criteria included a history of ocular surgery, the presence of media opacities interfering with retinal imaging, and a diagnosis of diabetes mellitus.

**Figure 1:**
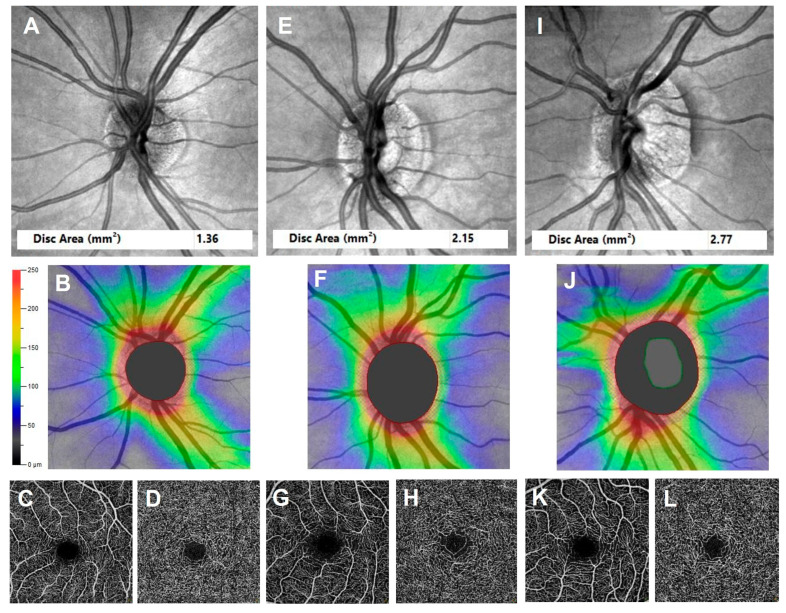
Examples for small, medium, and large optic discs based on BMOA in optical coherence tomography scans. Upper row: scanning laser ophthalmoscopy of optic discs. Middle row: thickness map of the corresponding optic discs. Lower row: three-by-three mm^2^ whole en face optical coherence tomography angiography of the corresponding macula; left: superficial capillary plexus (SCP); right: deep capillary plexus (DCP). (**A**–**D**): Small disc with a BMOA = 1.36 mm^2^, (**E**–**H**): medium disc with a BMOA = 2.15 mm^2^, (**I**–**L**): large disc with a BMOA = 2.77 mm^2^.

After confirming eligibility, OCT-A was performed using the RTVue XR Avanti system (AngioVue/RTVue-XR Avanti OCT, Optovue Inc., Fremont, CA, USA) in a dark, windowless room without pharmacologic pupil dilation. To minimize potential variations due to systemic physiological factors such as blood pressure or heart rate, participants were asked to rest for at least five minutes before image acquisition [25]. Angiographic macular scans covered a 3 × 3 mm^2^ area and provided en face images of the SCP, DCP, and FAZ. BMOA measurements were performed using the same system, focusing on the optic nerve head using 4.5 × 4.5 mm^2^ scans (Figure 1).

Only eyes for which both macular and optic disc scans were obtained consecutively within a five-minute interval were included in the analysis. In participants for whom both eyes met all inclusion criteria, the eye with the higher image quality was selected for analysis. If both eyes demonstrated identical image quality parameters, one eye was chosen at random. Each region (macula and optic disc) was imaged at least three times by an experienced examiner, and the scan with the highest quality index (QI) was selected for evaluation. If multiple images shared the same QI, one was chosen at random. Scans containing artefacts or incomplete data were excluded. Images were required to meet minimum quality thresholds of a signal strength index (SSI) ≥ 50 and a QI ≥ 6. OCT-A data were independently reviewed by two ophthalmologists experienced in this imaging technique; in case of discrepancies, a third examiner adjudicated.

In the absence of universally accepted normative cut-off values for OCT-based classification of optic disc size, subjects were allocated to three categories—small, medium, and large optic discs—using a quantile-based approach. Specifically, disc areas within the lowest and highest deciles were designated as small and large, respectively, while the intermediate 80% constituted the medium-sized group.

FD was automatically quantified using the AngioVue software algorithm (Version 2018.1.0.43), defined as the percentage of bright pixels relative to the total number of pixels within a scan area. FD values were computed for “whole en face” images representing overall vessel density in each retinal layer, as well as for distinct subregions within those layers. The internal segmentation algorithm differentiated between retinal layers and positioned the measurement grid over the foveal centre.

In total, 29 vascular parameters were extracted. Twelve parameters each described the macular SCP and DCP, including en face measurements and subregional analyses, such as parafoveal sectors and Early Treatment Diabetic Retinopathy Study (ETDRS) subfields. Additionally, five FAZ-related parameters were obtained: FAZ area, perimeter, acircularity index (ACI), and the FD within a 300 µm radius around the FAZ (FD-300 area density and length density). The ACI quantifies the deviation of FAZ shape from a perfect circle, with a value of 1.0 representing ideal circularity.

### Statistical Analysis

Data were entered and processed using Microsoft Excel (Version 16.71; Microsoft Corp., Redmond, WA, USA). Descriptive statistics are reported as mean ± standard deviation (SD). Eyes were categorized into optic disc area groups based on quantile distribution: the smallest 10% (*n* = 15; disc area < 1.48 mm^2^), the largest 10% (*n* = 15; disc area > 2.54 mm^2^), and the intermediate 80% (*n* = 121).

For the identification of associations between the optic disc area and macular parameters, a correlation analysis was performed. In the presence of skewed distributions, which were identified by visual inspection of histograms and calculation of skewness parameters, Spearman’s rank correlation coefficient was applied. Point estimates, 95% confidence intervals, and corresponding *p*-values were computed. Please note that *p*-values and confidence intervals for Spearman’s rank correlation coefficients might be unreliable in the presence of ties.

To identify further potential non-linear or non-monotonic relationships, all vascular parameters were additionally compared among three BMO-based optic disc size groups (the smallest 10%, largest 10%, and intermediate 80% of discs). Since the data were not normally distributed and measurements were unpaired, pairwise comparisons between the optic disc groups were performed using the Mann–Whitney U test. Results are presented as median values together with their interquartile ranges (25th–75th percentile). Associations were considered statistically significant if *p* < 0.05. Due to the exploratory character of this investigation, results should be interpreted with caution and validated in future confirmatory studies. All statistical analyses were conducted using R software (version 4.5.1) [26]. Plots were generated using the ggplot2 package [27].

## 3. Results

A total of 151 eyes from 151 participants were included in the final analysis. The participants’ ages ranged from 16 to 83 years. Sex distribution and laterality were evenly balanced. Neither the spherical equivalent nor the QI or SSI showed significant differences between the optic disc size groups. Baseline characteristics of the study population are summarized in Table 1.

### 3.1. Correlation Analysis

Correlation analysis revealed a significant negative association between BMOA and both DCP whole en face FD and FD-300 area density, indicating lower vascular density measures in eyes with larger BMOA values. The strongest association was observed for FD-300 area density, whereas the correlation with DCP whole en face FD was comparatively weak. No significant correlations were found between BMOA and SCP whole en face FD or FAZ area (Table 2).

**Figure 2:**
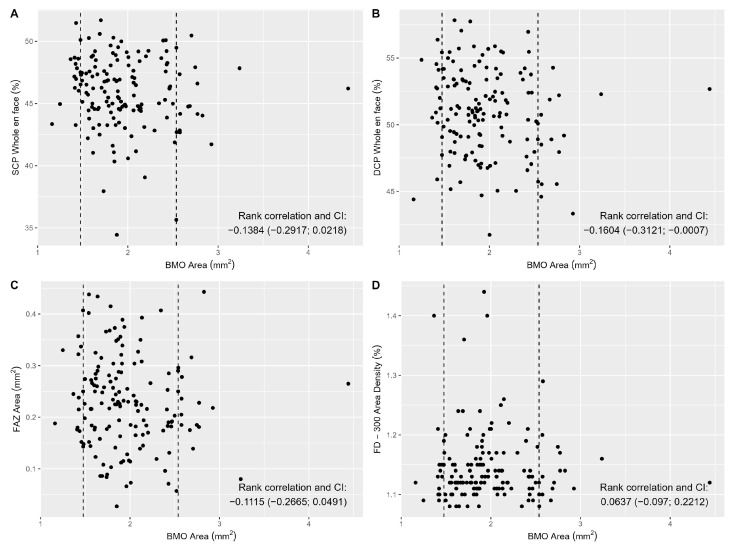
Scatter plots showing the relationship between BMOA and OCTA-derived vascular parameters. (**A**) Scatter plot of SCP whole en face and BMOA. (**B**) Scatter plot of DCP whole en face and BMOA. (**C**) Scatter plot of FAZ area and BMOA. (**D**) Scatter plot of FD-300 area density and BMOA. The plots visually illustrate the distribution of individual observations and the direction of the correlations reported in Table 2. A weak negative trend is apparent for DCP whole en face FD and is most pronounced for FD-300 area density, whereas no obvious association is observed for SCP whole en face FD or FAZ area. Note that dashed lines show bounds grouping by quantile distribution.

As illustrated in Figure 2, the scatter plots show substantial variability and only weak monotonic trends across most parameters. A visible tendency toward lower FD values with increasing BMOA was observed primarily for FD-300 area density and, to a lesser extent, DCP whole en face FD, whereas no clear trend was apparent for SCP whole en face FD or FAZ area.

Furthermore, statistical testing indicated a significant negative correlation between FD in several macular subsectors and BMOA (Table 3).

### 3.2. Optic Disc Groups (Quantile Division)

For the secondary hypothesis, comparisons were made between optic disc size groups based on BMO measurements between the smallest 10%, the largest 10%, and the remaining 80% of intermediate discs.

In this quantile-based analysis, FD values in the SCP were significantly higher in eyes with the smallest optic discs compared with those in the intermediate group across all subregions, except for the fovea and temporal parafoveal area. In contrast, none of the vascular parameters within the DCP demonstrated significant differences among these groups. When comparing the intermediate and largest disc groups, FD values in the DCP were significantly higher in the intermediate discs across all subregions except for the foveal, temporal parafoveal, and nasal parafoveal areas. No significant differences in SCP parameters were observed among these cohorts. Furthermore, when comparing the smallest and largest optic discs, FD values in both the SCP and DCP were significantly higher in eyes with the smallest discs across all subregions, except for the fovea and the superior and temporal parafoveal regions (Table 4).

Notable group differences were also evident in the characteristics of the FD-300 measurements. The FD-300 length was significantly greater in eyes with the smallest optic discs compared with both the intermediate and largest groups. Similarly, the FD-300 density was significantly higher in the smallest discs compared with the largest ones. By contrast, no significant differences were found in FAZ parameters, including FAZ area, perimeter, or ACI, between any of the optic disc size groups (Table 5).

## 4. Discussion

The findings of this retrospective study can be summarized as follows. Statistical analysis revealed a significant inverse relationship between macular FD in both the SCP and DCP as well as BMOA. When stratified by disc size quantiles, macular FD in the SCP and DCP was significantly higher in eyes with small optic discs compared to those with large discs. In contrast, foveal FD as well as morphologic FAZ parameters appeared unchanged. By taking optic disc size into account, this study contributes additional normative data for macular FD.

To our knowledge, this is the first study to investigate the association between macular FD and BMOA, as well as FAZ parameters and BMOA, using OCT-A in a large cohort of healthy individuals. The negative correlation seen between FD in both SCP and DCP as well as BMOA in both regression analysis and quantile-based analysis indicates that eyes with smaller optic discs tend to exhibit higher macular FD values in both inner retinal vascular plexuses.

Since this is the first study to explore the relationship between macular angiographic metrics with structural optic disc parameters, directly comparable data are not available. One of the most analogous works to date is that of Fernández-Vigo et al., who analyzed the correlation between macular OCT-A parameters and optic disc characteristics in a large healthy cohort of 346 right eyes [28]. In both the SCP as well as DCP, they found no association between macular and papillary FD; however, their analysis was limited to angiography parameters only and did not consider structural optic disc data, such as optic disc area.

Savini et al. adopted a structural approach, investigating optic disc area in relation to RNFL thickness [10], and reported a positive correlation between RNFL thickness and optic disc size in healthy eyes. Similar findings were reported by Seo et al. in 168 eyes using a CSLO-based approach [9]. To align our results with these observations, one possible explanation is that the higher macular FD observed in eyes with smaller optic discs may reflect a compensatory response to the lower RNFL thickness associated with smaller disc size. However, this interpretation is challenged by evidence showing that in glaucoma—where RNFL thickness is reduced—macular FD decreases rather than increases [29,30,31]. Moreover, the negative correlation in our study was not limited to the SCP, which primarily supplies the RNFL, but was equally present in the DCP, which predominantly nourishes bipolar cells and photoreceptors.

Since visual function, as reflected by visual acuity (VA), did not differ significantly between the groups, the observed association between optic disc area and macular FD appears to represent a structural, physiologically compensatory adaptation rather than a pathological finding. A structural parameter that may contribute to the underlying mechanism in a similar way is axial length (AL). Several authors have demonstrated a positive correlation between AL and optic disc size. Oliveira et al. examined 281 eyes of healthy adults using CSLO and A-scan ultrasonography and showed that larger discs were associated with longer AL [32]. Similarly, Samarawickrama et al., who studied 4118 children, found a positive association between optic disc area and AL independently of spherical equivalent [33]. More recent studies report a negative correlation between AL and macular FD: Wen et al. observed lower macular FD in eyes with longer AL in 75 healthy participants [34], and Youssef et al. confirmed this inverse relationship in 112 eyes from 56 healthy subjects [35]. Taken together, these findings could suggest that disc enlargement and ocular elongation may reduce the spatial density of central photoreceptors, thereby lowering metabolic demand and resulting in reduced perfusion requirements. Consequently, it cannot be excluded that AL may partially mediate the observed association between BMOA and macular FD. Since AL measurements were not available in the present retrospective cohort, the independence of this relationship cannot be definitively established and should be investigated in future studies incorporating comprehensive ocular biometry.

Optic disc size represents a fundamental factor in the evaluation of the ONH. Traditionally, optic disc assessment relied on slit-lamp biomicroscopy or fundus photography, both of which provide only approximate estimations of disc size. Advances in retinal imaging over recent decades have enabled reproducible, quantitative measurements of optic disc parameters. CSLO has long been used to characterize disc morphology, but the advent of OCT allows for three-dimensional ONH reconstruction and more anatomically accurate assessments. Importantly, OCT enables precise identification of BMO, the anatomical landmark defining the optic disc border. Consequently, BMOA has become a reliable structural parameter for evaluating ONH morphology and disc size [36].

The literature remains inconsistent regarding the determination of optic disc size using HRT versus OCT. Cazana et al. reported systematic differences between CSLO-derived disc area and OCT-based BMOA and proposed a conversion formula to harmonize both modalities: CSLO × 0.73 + 0.3 = BMOA [22]. In contrast, Scheuble et al. found no significant correlation between HRT-derived disc area and BMOA in their cohort of 216 glaucoma patients [37]. Such discrepancies may arise from disease-related alterations of the ONH. Given the absence of universally accepted BMOA-based thresholds for micro-, normal, and macrodiscs, the present study applied a 10% quantile approach to specifically examine the extreme disc sizes.

The use of a quantile-based approach allows for focused evaluation of extreme disc sizes. Conventional OCT devices typically classify measurements as normal, borderline, or pathological based on reference databases. Recently, Ramachandran et al. have shown that current databases may not accurately reflect the diversity of human ONH anatomy, potentially leading to misclassification of extreme disc sizes [38]. Similarly, Artes et al. explain that extreme disc sizes are underrepresented in these normative databases due to the limitations of linear regression in modelling the distribution of extremes, and propose quantile regression as a solution, as it provides more accurate normative limits [39]. In line with this, our group demonstrated that differences in RNFL thickness between extreme optic disc sizes became more pronounced when analyzed in a groupwise manner, whereas conventional analyses across the full cohort tended to obscure such variations [14]. In a similar manner, the study at hand demonstrates substantial differences between the extreme disc groups, while differences relative to the median disc group were comparatively smaller. These findings underscore the importance of investigating these edge cases, as they may harbour structural or perfusion-related processes that are not fully understood. Consequently, future studies specifically targeting extreme optic disc sizes are needed to further elucidate these mechanisms and refine normative OCT-A references.

Taken together, these findings highlight the importance of considering optic disc size—quantified by BMOA—when interpreting macular OCT-A metrics. Even though the observed variations in FD appear to reflect physiological structural differences rather than functional impairment, they may nonetheless influence quantitative perfusion measurements and could therefore be relevant in clinical decision-making, particularly when evaluating patients at the extremes of disc size. If confirmed by future studies that account for ocular biometry, incorporating BMOA into OCT-A assessment may help clinicians distinguish true pathological perfusion deficits from normal anatomical variability. Future studies should further explore this structure–flow relationship in disease states to determine whether the compensatory patterns observed in healthy eyes persist or break down once neurovascular integrity is compromised.

### Limitations

Several limitations should be acknowledged.

First, due to the retrospective design of the present study, no causal inferences or prospective estimations can be made. Future longitudinal studies could help clarify whether optic disc size has a causal influence on retinal microcirculation or whether it simply reflects interindividual anatomical variation.

Second, AL, which has been shown to correlate with both optic disc size and macular OCT-A parameters, was not directly measured in the present study. Therefore, it cannot be entirely excluded that the observed associations between BMOA and macular FD may in part reflect effects related to ocular elongation. However, refractive error—serving as an indirect surrogate for AL—did not differ significantly between the optic disc size groups, suggesting that relevant differences in AL are unlikely to have driven the observed findings.

Third, as reported by various authors, ethnicity may significantly influence both optic disc morphology [40,41] and OCT-A-derived vascular parameters [42,43]. Since this analysis exclusively included individuals of Caucasian descent, the results may not be directly generalizable to populations with different ethnic backgrounds.

Finally, although this study comprises, to our knowledge, the largest cohort analyzed regarding this research question to date, the overall sample size remains limited and thus restricts the generalizability of our conclusions. To validate and extend these findings, larger multi-centre studies involving more diverse populations will be necessary to confirm the observed correlations.

## 5. Conclusions

In summary, this study demonstrated a significant inverse relationship between macular FD in both SCP and DCP and BMOA. Conversely, foveal FD, as well as FAZ area and perimeter, were not influenced by optic disc size in this cohort of healthy individuals. The provision of reference values for FD and FAZ parameters across different optic disc size groups may facilitate the differentiation of physiological variations from early pathological alterations in clinical assessments. Moreover, these findings emphasize the importance of considering optic disc morphology when interpreting quantitative OCT-A metrics to enhance diagnostic precision in both research and clinical settings.

## Figures and Tables

**Table 1:** General patient characteristics. Values are reported as absolute numbers and the median (25th and 75th quartile).

*n* (Eyes)	151
*n* (patients)	151
Age (years)	32 (26; 38)
Gender (M:F)	75 (50%):76 (50%)
*n* (eyes) according to optic disc size (quantile division):	
Largest (2.54–4.44 mm^2^)	15 (10%)
Intermediate (1.48–2.54 mm^2^)	121 (80%)
Smallest (1.16–1.49 mm^2^)	15 (10%)
Study eye (R:L)	79 (52%):72 (48%)
Median visual acuity (logMAR)	0.00 (0.00; 0.00)
Median spherical equivalent	−0.125 (−1.5; 0.00)
Per group:	
Largest	0.00 (−0.38; 0.63)
Intermediate	−0.25 (−1.50; 0.00)
Smallest	−0.375 (−1.94; 0.00)
Median QI	8.00 (8.00; 9.00)
Per group:	
Largest	8.00 (8.00; 9.00)
Intermediate	8.00 (7.00; 8.00)
Smallest	8.00 (8.00; 9.00)
Median SSI:	70.15 (65.95; 75.36)
Per group	
Largest	67.89 (64.92; 72.56)
Intermediate	69.87 (66.29; 75.64)
Smallest	71.48 (67.92; 74.02)

*n* = number; M = male; F = female; R = right; L = left; logMAR = logarithm of the minimum angle of resolution; QI = quality index; SSI = signal strength index.

**Table 2:** Estimates and 95% confidence intervals for correlation analysis between FD values and BMOA. *p*-values < 0.05 are highlighted in bold.

	Estimate	Lower 95% CI	Upper 95% CI	*p*
SCP Whole en face (%)	−0.14	−0.29	0.02	0.09
DCP Whole en face (%)	−0.16	−0.31	−0.00	**0.05**
FAZ area (mm^2^)	−0.11	−0.26	0.05	0.17
FD-300 Area Density (%)	−0.26	−0.40	−0.10	**<0.01**

SCP = superficial macular capillary plexus, DCP = deep macular capillary plexus, FAZ = foveal avascular zone, mm = millimetres, FD-300 = flow density of the capillaries adjacent to the FAZ, CI = confidence interval.

**Table 3:** Estimates and 95% confidence intervals for correlation analysis between subregional FD values and BMOA. *p*-values < 0.05 are highlighted in bold.

		Estimate	Lower 95% CI	Upper 95% CI	*p*
SCP (%)	Whole en face sup hem	−0.11	−0.26	0.05	0.19
	Whole en face inf hem	−0.18	−0.33	−0.02	**0.03**
	Whole en face EDTRS	−0.15	−0.30	0.01	0.07
	Fovea	0.07	−0.09	0.23	0.37
	Parafovea	−0.18	−0.33	−0.02	**0.03**
	Parafovea sup hem	−0.14	−0.30	0.02	0.08
	Parafovea inf hem	−0.19	−0.34	−0.03	**0.02**
	Parafovea temporal	−0.11	−0.26	0.05	0.18
	Parafovea sup	−0.16	−0.31	0.00	**0.05**
	Parafovea nasal	−0.14	−0.30	0.02	0.08
	Parafovea inf	−0.24	−0.38	−0.08	**<0.01**
DCP (%)	Whole en face sup hem	−0.15	−0.30	0.01	0.06
	Whole en face inf hem	−0.16	−0.31	0.00	**0.05**
	Whole en face EDTRS	−0.16	−0.31	0.00	0.05
	Fovea	0.07	−0.09	0.23	0.38
	Parafovea	−0.20	−0.34	−0.04	**0.02**
	Parafovea sup hem	−0.19	−0.34	−0.04	**0.02**
	Parafovea inf hem	−0.19	−0.35	−0.04	**0.02**
	Parafovea temporal	−0.17	−0.32	−0.01	**0.04**
	Parafovea sup	−0.16	−0.32	−0.01	**0.04**
	Parafovea nasal	−0.23	−0.38	−0.08	**<0.01**
	Parafovea inf	−0.18	−0.33	−0.02	**0.02**
FAZ	Perimeter (mm)	−0.11	−0.27	0.05	0.16
	ACI	0.06	−0.10	0.22	0.43
	FD-300 Area Length (%)	−0.20	−0.35	−0.04	**0.01**

SCP = superficial macular capillary plexus, DCP = deep macular capillary plexus, FAZ = foveal avascular zone, sup = superior, inf = inferior, hem = hemisphere, EDTRS = Early Treatment Diabetic Retinopathy Study, parafovea = area surrounding the fovea, mm = millimetres, ACI = acircularity index, CI = confidence interval.

**Table 4:** Results of statistical comparison between optic disc cohorts in 10% quantile divisions. FD values are presented as the median (25% quartile; 75% quartile). *p*-values < 0.05 are highlighted in bold.

		Smallest	Intermediate	*p*	Intermediate	Largest	*p*	Smallest	Largest	*p*
SCP	Whole	47.51 (46.15; 48.59)	45.95 (44.50; 47.64)	**0.03**	45.95 (44.50; 47.64)	44.76 (43.52; 46.99)	0.43	47.51 (46.15; 48.59)	44.76 (43.52; 46.99)	**0.02**
	-sup hem	47.33 (46.06; 48.63)	45.69 (43.99; 47.85)	**0.03**	45.69 (43.99; 47.85)	44.76 (43.52; 47.16)	0.55	47.33 (46.06; 48.63)	44.76 (43.52; 47.16)	**0.04**
	-inf hem	47.88 (45.83; 48.81)	45.94 (44.57; 47.73)	**0.03**	45.94 (44.57; 47.73)	44.42 (43.96; 46.55)	0.35	47.88 (45.83; 48.81)	44.42 (43.96; 46.55)	**0.02**
	-EDTRS	47.02 (45.60; 47.75)	45.50 (43.68; 47.12)	**0.03**	45.50 (43.68; 47.12)	44.02 (43.07; 46.45)	0.37	47.02 (45.60; 47.75)	44.02 (43.07; 46.45)	**0.02**
	Fovea	20.36 (14.68; 22.35)	19.98 (16.58; 24.03)	0.36	19.98 (16.58; 24.03)	17.96 (15.16; 23.23)	0.56	20.36 (14.68; 22.35)	17.96 (15.16; 23.23)	0.85
	Parafovea	50.20 (48.99; 51.60)	48.82 (46.75; 50.41)	**0.01**	48.82 (46.75; 50.41)	47.64 (46.33; 49.50)	0.40	50.20 (48.99; 51.60)	47.64 (46.33; 49.50)	**<0.01**
	-sup hem	50.52 (48.40; 51.40)	48.73 (46.49; 50.45)	**<0.01**	48.73 (46.49; 50.45)	47.11 (46.27; 49.30)	0.51	50.52 (48.40; 51.40)	47.11 (46.27; 49.30)	**<0.01**
	-inf hem	51.10 (48.96; 52.03)	49.00 (46.99; 50.84)	**0.02**	49.00 (46.99; 50.84)	47.90 (44.49; 49.39)	0.34	51.10 (48.96; 52.03)	47.90 (44.49; 49.39)	**0.01**
	-temporal	48.01 (46.73; 50.96)	47.01 (45.38; 49.10)	0.09	47.01 (45.38; 49.10)	47.03 (45.90; 48.75)	0.91	48.01 (46.73; 50.96)	47.03 (45.90; 48.75)	0.15
	-sup	51.20 (49.56; 52.38)	49.79 (47.26; 51.59)	**<0.01**	49.79 (47.26; 51.59)	48.11 (46.16; 50.85)	0.38	51.20 (49.56; 52.38)	48.11 (46.16; 50.85)	**<0.01**
	-nasal	49.58 (48.86; 51.42)	48.31 (45.69; 50.31)	**0.01**	48.31 (45.69; 50.31)	47.81 (46.40; 48.60)	0.94	49.58 (48.86; 51.42)	47.81 (46.40; 48.60)	**0.02**
	-inf	51.63 (50.27; 56.60)	50.49 (48.50; 52.34)	**<0.01**	50.49 (48.50; 52.34)	49.01 (45.95; 50.86)	0.15	51.63 (50.27; 56.60)	49.01 (45.95; 50.86)	**<0.01**
DCP	Whole	52.01 (50.34; 54.34)	51.02 (49.07; 53.39)	0.60	51.02 (49.07; 53.39)	48.90 (45.64; 52.05)	**0.03**	52.01 (50.34; 54.34)	48.90 (45.64; 52.05)	**0.04**
	-sup hem	52.34 (49.80; 54.50)	51.26 (48.65; 53.18)	0.73	51.26 (48.65; 53.18)	48.69 (45.61; 51.59)	**0.02**	52.34 (49.80; 54.50)	48.69 (45.61; 51.59)	**0.04**
	-inf hem	52.10 (50.88; 54.01)	51.10 (48.92; 53.62)	0.48	51.10 (48.92; 53.62)	48.31 (46.05; 52.18)	**0.06**	52.10 (50.88; 54.01)	48.31 (46.05; 52.18)	**0.04**
	-EDTRS	51.74 (50.25; 54.02)	51.16 (49.34; 53.19)	0.54	51.16 (49.34; 53.19)	49.07 (47.70; 51.59)	**0.04**	51.74 (50.25; 54.02)	49.07 (47.70; 51.59)	**0.04**
	Fovea	36.93 (31.45; 38.98)	37.12 (32.26; 41.45)	0.32	37.12 (32.26; 41.45)	34.38 (31.12; 39.81)	0.64	36.93 (31.45; 38.98)	34.38 (31.12; 39.81)	0.76
	Parafovea	54.50 (51.94; 55.89)	53.18 (50.74; 55.36)	0.44	53.18 (50.74; 55.36)	51.41 (49.28; 52.81)	**0.03**	54.50 (51.94; 55.89)	51.41 (49.28; 52.81)	**0.03**
	-sup hem	53.57 (51.33; 56.45)	53.11 (50.57; 55.31)	0.52	53.11 (50.57; 55.31)	51.24 (49.49; 52.44)	**0.01**	53.57 (51.33; 56.45)	51.24 (49.49; 52.44)	**0.03**
	-inf hem	54.30 (52.20; 55.47)	53.24 (51.14; 55.27)	0.38	53.24 (51.14; 55.27)	51.60 (49.00; 53.11)	**0.05**	54.30 (52.20; 55.47)	51.60 (49.00; 53.11)	**0.03**
	-temporal	54.48 (51.99; 56.88)	53.54 (51.65; 55.65)	0.36	53.54 (51.65; 55.65)	51.75 (50.03; 54.30)	0.10	54.48 (51.99; 56.88)	51.75 (50.03; 54.30)	0.06
	-sup	53.15 (48.82; 55.96)	52.87 (50.13; 54.94)	0.86	52.87 (50.13; 54.94)	50.61 (47.99; 52.13)	**0.01**	53.15 (48.82; 55.96)	50.61 (47.99; 52.13)	0.07
	-nasal	55.16 (52.20; 56.34)	53.36 (51.33; 55.55)	0.15	53.36 (51.33; 55.55)	52.37 (50.40; 54.41)	0.08	55.16 (52.20; 56.34)	52.37 (50.40; 54.41)	**0.02**
	-inf	53.38 (51.71; 55.17)	53.27 (50.46; 55.45)	0.56	53.27 (50.46; 55.45)	50.48 (46.93; 52.66)	**0.03**	53.38 (51.71; 55.17)	50.48 (46.93; 52.66)	**0.03**

SCP = superficial macular capillary plexus, DCP = deep macular capillary plexus, sup = superior, inf = inferior, hem = hemisphere, EDTRS = Early Treatment Diabetic Retinopathy Study, parafovea = area surrounding the fovea.

**Table 5:** Results of statistical comparison between optic disc cohorts in 10% quantile divisions. FAZ values are presented as the median (25% quartile; 75% quartile). *p*-values < 0.05 are highlighted in bold.

Parameter	Smallest	Intermediate	*p*	Intermediate	Largest	*p*	Smallest	Largest	*p*
FAZ area (mm^2^)	0.24 (0.18; 0.33)	0.23 (0.17; 0.28)	0.31	0.23 (0.17; 0.28)	0.22 (0.18; 0.27)	0.93	0.24 (0.18; 0.33)	0.22 (0.18; 0.27)	0.49
Perimeter (mm)	1.99 (1.71; 2.27)	1.90 (1.68; 2.11)	0.17	1.90 (1.68; 2.11)	1.92 (1.72; 2.06)	0.82	1.99 (1.71; 2.27)	1.92 (1.72; 2.06)	0.41
ACI	1.13 (1.11; 1.15)	1.13 (1.11; 1.16)	0.90	1.13 (1.11; 1.16)	1.13 (1.11; 1.17)	0.90	1.13 (1.11; 1.15)	1.13 (1.11; 1.17)	0.80
FD-300 Area Density (%)	50.58 (48.85; 52.60)	49.30 (46.92; 51.49)	0.06	49.30 (46.92; 51.49)	48.64 (46.43; 50.08)	0.20	50.58 (48.85; 52.60)	48.64 (46.43; 50.08)	**0.02**
FD-300 Area Length (%)	18.73 (17.82; 18.88)	17.77 (16.57; 18.69)	**0.03**	17.77 (16.57; 18.69)	16.87 (16.27; 17.65)	0.20	18.73 (17.82; 18.88)	16.87 (16.27; 17.65)	**0.02**

FAZ = foveal avascular zone, mm = millimetres, ACI = acircularity index, FD-300 = flow density of the capillaries adjacent to the FAZ.

## Data Availability

The original contributions presented in this study are included in the article. Further inquiries can be directed to the corresponding author.
